# The Tatton-Brown-Rahman Syndrome: A clinical study of 55 individuals with *de novo* constitutive *DNMT3A *variants

**DOI:** 10.12688/wellcomeopenres.14430.1

**Published:** 2018-04-23

**Authors:** Katrina Tatton-Brown, Anna Zachariou, Chey Loveday, Anthony Renwick, Shazia Mahamdallie, Lise Aksglaede, Diana Baralle, Daniela Barge-Schaapveld, Moira Blyth, Mieke Bouma, Jeroen Breckpot, Beau Crabb, Tabib Dabir, Valerie Cormier-Daire, Christine Fauth, Richard Fisher, Blanca Gener, David Goudie, Tessa Homfray, Matthew Hunter, Agnete Jorgensen, Sarina G. Kant, Cathy Kirally-Borri, David Koolen, Ajith Kumar, Anatalia Labilloy, Melissa Lees, Carlo Marcelis, Catherine Mercer, Cyril Mignot, Kathryn Miller, Katherine Neas, Ruth Newbury-Ecob, Daniela T. Pilz, Renata Posmyk, Carlos Prada, Keri Ramsey, Linda M. Randolph, Angelo Selicorni, Deborah Shears, Mohnish Suri, I. Karen Temple, Peter Turnpenny, Lionel Van Maldergem, Vinod Varghese, Hermine E. Veenstra-Knol, Naomi Yachelevich, Laura Yates, Clinical Assessment of the Utility of Sequencing and Evaluation as a Service (CAUSES) Research Study, Deciphering Developmental Disorders (DDD) Study, Nazneen Rahman

**Affiliations:** 1 Division of Genetics and Epidemiology, Institute of Cancer Research, London, UK; 2 South West Thames Regional Genetics Service, St George’s University Hospitals NHS Foundation Trust, London, UK; 3 St George’s University of London, London, UK; 4 Department of Clinical Genetics, Copenhagen University Hospital, Copenhagen, Denmark; 5 Human Genetics and Genomic Medicine, Faculty of Medicine, University of Southampton, Southhampton, UK; 6 Department of Clinical Genetics, Leiden University Medical Centre, Leiden, The Netherlands; 7 Department of Clinical Genetics, Chapel Allerton Hospital, Leeds, UK; 8 Elver Intellectual Disability Centre, Nieuw Wehl, The Netherlands; 9 Center for Human Genetics, University Hospitals and KU Leuven, Leuven, Belgium; 10 Genetics Department, Children's Hospitals and Clinics of Minneapolis, Minneapolis, MN, USA; 11 Northern Ireland Regional Genetics Centre, Clinical Genetics Service, Belfast City Hospital, Belfast, UK; 12 INSERM UMR1163, IMAGINE Institute affiliate, Paris, France; 13 Division of Human Genetics, Medical University Innsbruck, Innsbruck, Austria; 14 Teesside Genetics Unit, The James Cook University Hospital, Middlesbrough, UK; 15 Department of Genetics, Cruces University Hospital, Biocruces Health Research Institute, centro de Investigacion Biomedica en Red de Enfermedades Raras (CIBERER), Basque Country, Spain; 16 Department of Human Genetics, Ninewells Hospital and Medical School, Dundee, UK; 17 Monash Genetics, Monash Health, Melbourne, Australia; 18 Department of Paediatrics, Monash University, Melbourne, Australia; 19 Division of Child and Adolescent Health, Department of Medical Genetics, University Hospital of North Norway, Tromsø, Norway; 20 Department of Health, Genetic Services of Western Australia, Subiaco, Australia; 21 Department of Human Genetics and Donders Institute for Brain, Cognition and Behaviour, Radboud University Medical Center, Nijmegen, The Netherlands; 22 North East Thames Regional Genetics Service and Department of Clinical Genetics, Great Ormond Street Hospital, London, UK; 23 Department of Pediatrics, University of Cincinnati, College of Medicine, Division of Genetics, Cincinnati Children’s Hospital Medical Center, Cincinnati, OH, USA; 24 Hospital Internacional de Colombia, Floridablanca, Colombia; 25 Département de Génétique and Centre de Référence Déficiences Intellectuelles de Causes Rares, Assistance Publique – Hôpitaux de Paris , Paris, France; 26 Albany Medical Center, New York, NY, USA; 27 Genetic Health Service New Zealand, Wellington, New Zealand; 28 University Hospitals Bristol NHS Trust/University of Bristol, Bristol, UK; 29 West of Scotland Clinical Genetics Service, Queen Elizabeth University Hospital,, Glasgow, UK; 30 Department of Clinical Genetics, Podlaskie Medical Center, Bialystok, Poland; 31 Department of Perinatology and Obstetrics, Medical University in Bialystok, Bialystok, Poland; 32 Center for Rare Childhood Disorders, Translational Genomics Research Institute, Phoenix, AZ, USA; 33 Division of Medical Genetics, Children's Hospital Los Angeles, University of Southern California/ Keck School of Medicine, Los Angeles, CA, USA; 34 UOC Pediatria ASST Laraina, Como, Italy; 35 Oxford Centre for Genomic Medicine, Oxford University Hospitals NHS Foundation Trust, Oxford, UK; 36 Nottingham Clinical Genetics Service, City Hospital Campus, Nottingham University Hospitals NHS Trust, Nottingham, UK; 37 Peninsula Clinical Genetics, University of Exeter Medical School, Royal Devon & Exeter NHS Foundation Trust, Exeter, UK; 38 Centre de Génétique Humaine and Integrative and Cognitive Neuroscience Research Unit EA481, Besançon, Besançon, France; 39 Institute of Medical Genetics, University Hospital of Wales, Cardiff, UK; 40 Department of Genetics, University Medical Center Groningen, University of Groningen, Groningen, The Netherlands; 41 Clinical Genetics Services, New York University Hospitals Center, New York University, New York, NY, USA; 42 Cancer Genetics Unit, Royal Marsden NHS Foundation Trust, London, UK

**Keywords:** DNMT3A, Tatton-Brown-Rahman, overgrowth, intellectual disability

## Abstract

Tatton-Brown-Rahman syndrome (TBRS; OMIM 615879), also known as the DNMT3A-overgrowth syndrome, is an overgrowth intellectual disability syndrome first described in 2014 with a report of 13 individuals with constitutive heterozygous *DNMT3A* variants. Here we have undertaken a detailed clinical study of 55 individuals with *de novo* *DNMT3A *variants, including the 13 previously reported individuals. An intellectual disability and overgrowth were reported in >80% of individuals with TBRS and were designated major clinical associations. Additional frequent clinical associations (reported in 20-80% individuals) included an evolving facial appearance with low-set, heavy, horizontal eyebrows and prominent upper central incisors; joint hypermobility (74%); obesity (weight ³2SD, 67%); hypotonia (54%); behavioural/psychiatric issues (most frequently autistic spectrum disorder, 51%); kyphoscoliosis (33%) and afebrile seizures (22%). One individual was diagnosed with acute myeloid leukaemia in teenage years. Based upon the results from this study, we present our current management for individuals with TBRS

## Introduction

Tatton-Brown-Rahman syndrome (TBRS; OMIM 615879), also known as the DNMT3A-overgrowth syndrome, is an overgrowth intellectual disability (OGID) syndrome first described in 2014 with a report of 13 individuals with *de novo* heterozygous *DNMT3A* variants ^1, 2^. Subsequently, a further 22 individuals with TBRS have been reported ^3– 9^.

In this report we have undertaken a detailed clinical evaluation of 55 individuals with *de novo DNMT3A* variants, including the 13 individuals we first reported in 2014. We have expanded and clarified the TBRS phenotype, delineating major and frequent clinical associations, which has informed our management of individuals with this new OGID syndrome.

## Methods

The study was approved by the London Multicentre Research Ethics Committee (MREC MREC/01/2/44). Patients were identified through Clinical Genetics Services worldwide and written informed consent was obtained from all participating individuals and/or parents. Photographs, with accompanying written informed consent to publish, were requested from all participants and received from the families of 41 individuals. Detailed phenotype data were collected through a standardized clinical proforma, a *DNMT3A* specific clinical proforma and clinical review by one of the authors. Growth parameter standard deviations were calculated with reference to UK90 growth data ^10^.

The degree of intellectual disability was defined in relation to educational support as a child and living impairment as an adult:

- an individual with a mild intellectual disability typically had delayed milestones but would attend a mainstream school with some support and live independently, with support, as an adult;
- an individual with a moderate intellectual disability typically required high level support in a mainstream school or special educational needs schooling and would live with support as an adult;
- an individual with a severe intellectual disability typically required special educational needs schooling, had limited speech, and would not live independently as an adult.

**Figure 1.**
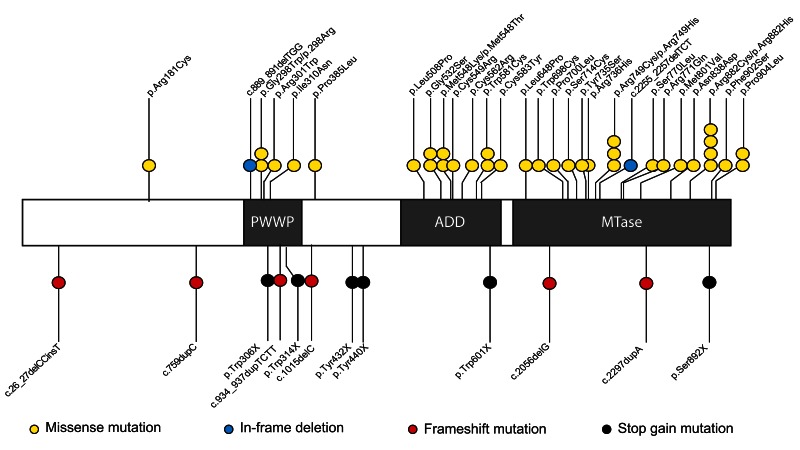
DNMT3A and the positions and types of variants with protein truncating variants shown below the protein (black and red lollipops) and missense variants and inframe deletions (yellow and blue lollipops) shown above the protein. Whole gene deletions and the splice site variant are not shown on this figure. The three DNMT3A domains are shaded in grey: the proline-tryptophan-tryptophan-proline (PWWP) domain, the ATRX-Dnmt3-Dnmt3L (ADD) domain and the Methyltransferase (MTase) domain.

55 individuals were included with a range of *de novo* heterozygous *DNMT3A* variants: missense variants (36 individuals with 30 different variants); stop gain variants (six individuals); frameshift variants (six individuals); whole gene deletions (four individuals including identical twins (COG1961 and COG2006)); in-frame deletions (two individuals) and a splice site variant (one individual, Figure 1, Table 1). Computational tools predicted all 30 missense variants to be deleterious ( Mutation Taster2 and SIFT (version 6.2.1), Supplementary Table 1) and the splice site variant was predicted to disrupt normal splicing. Importantly, some of the variants are common in the general population due to age-related clonal haematopoiesis, limiting the utility of databases such as gnomAD in *DNMT3A* variant pathogenicity stratification ( Supplementary Table 1) ^11, 12^.

**Table 1.** Table of all individuals with TBRS and their associated phenotypes including growth and cognitive profiles.

Case number	Variant type	Nucleotide change	Protein change	Inheritance	BW/ SD	BHC/ SD	BL/ SD	Age/ yrs	Ht/ SD	HC/ SD	Wt/ SD	ID	Behavioural issues	Joint hyper mobility	Hypotonia	Kyphoscoliosis	Afebrile seizures	Other clinical issues
COG1849	frameshift	c.26_27delinsT		de novo	1.0	nk	nk	10.0	5.1	nk	nk	mod	ASD	no	yes	no	yes	Multiple fungal and viral infections, precocious puberty, leg length discrepancy
COG1919	missense	c.541C>T	p.(Arg181Cys)	de novo	nk	nk	nk	11.3	3.1	1.6	2.8	mod	no	no	no	no	no	Pre-auricular skin tags, 5th toe nail hypoplasia
COG2017	frameshift	c.759dupC		de novo	-0.4	nk	nk	7.7	3.9	2.2	3.3	mod	no	yes	yes	no	no	CAL macules, soft skin
COG0274	in-frame deletion	c.889_891delTGG		de novo	3.3	nk	1.7	18.0	3.0	2.7	nk	mod	no	nk	yes	no	yes	
COG1843	missense	c.892G>T	p.(Gly298Trp)	de novo	1.6	nk	4.4	12.1	4.1	2.2	3.9	mod	ASD, anxiety	yes	yes	no	no	Arachnoid cyst, hypospadias
COG2008/ DDD260414	missense	c.892G>A	p.(Gly298Arg)	de novo	2.1	2.8	nk	18.0	0.2	0.7	2.9	mod	Anxiety	yes	no	yes	no	Myopia (-3D)
COG2019/ DDD293780	missense	c.901C>T	p.(Arg301Trp)	de novo	nk	nk	nk	9.3	2.1	2.1	1.3	mild	no	yes	no	no	no	
COG1963	stop gain	c.918G>A	p.(Trp306X)	de novo	1.5	1.2	nk	6.2	2.7	4.0	1.9	sev	ASD, regression	nk	yes	no	yes	Seizures
COG1770	missense	c.929T>A	p.(Ile310Asn)	de novo	2.2	2.8	2.7	10.3	3.8	3.3	3.3	sev	ASD, compulsive eating	yes	yes	yes	yes	Ventriculomegaly and Chiari malformation, multiple renal cysts, multiple urinary tract infections, constipation, lumbar haemangioma
COG1670	frameshift	c.934_937dupTCTT		de novo	3.6	nk	nk	20.5	3.2	2.8	2.8	sev	Temper tantrums, aggressive, Psychosis (paranoid hallucinations)	no	no	no	no	
COG1962/ DDD271500	stop gain	c.941G>A	p.(Trp314X)	de novo	0.7	nk	nk	5.0	2.1	0.5	2.2	mod	no	no	no	no	no	
COG1974	frameshift	c.1015delC		de novo	1.4	1.6	0.4	10.0	2.0	1.4	2.1	mod	no	no	no	no	no	
COG1998	missense	c.1154C>T	p.(Pro385Leu)	de novo	-0.7	2.3	1.4	5.2	3.1	0.8	2.1	mod	ASD	yes	yes	yes	no	
COG1916	stop gain	c.1296C>G	p.(Tyr432X)	de novo	2.9	4.4	3.6	21.0	3.9	0.6	3.2	mod	ASD	yes	no	yes	no	AVNRT, mitral regurgitation, pectus carinatum, amblyopia, photophobia
COG2007/ DDD294475	stop gain	c.1320G>A	p.(Trp440X)	de novo	1.8	nk	nk	10.5	3.2	2.8	1.3	mod	no	yes	no	no	no	Cryptorchidism
COG1925	missense	c.1523T>C	p.(Leu508Pro)	de novo	2.8	6.5	3.8	6.3	4.0	3.7	4.4	mild	ASD	yes	yes	yes	no	Cryptorchidism
COG0141	missense	c.1594G>A	p.(Gly532Ser)	de novo	2.2	nk	nk	25.0	2.3	2.9	4.5	mod	ASD	no	no	no	no	
COG1995	missense	c.1594G>A	p.(Gly532Ser)	de novo	3.9	nk	nk	22.0	2.9	3.6	3.0	mild	ASD	yes	no	no	no	
COG0422	missense	c.1643T>A	p.(Met548Lys)	de novo	1.3	1.6	nk	15.3	1.4	3.4/12.8 yrs	3.4	sev	Aggression	yes	yes	no	no	Atrial septal defect
COG2009/ DDD282776	missense	c.1643T>C	p.(Met548Thr)	de novo	1.7	nk	nk	15.3	1.7	3.4	1.9	sev	ASD	yes	yes	no	yes	Umbilical hernia, early puberty, cryptorchidism
COG1288	missense	c.1645T>C	p.(Cys549Arg)	de novo	1.1	1.6	2.6	17.9	1.6	3.6	2.6	mod	no	yes	yes	yes	no	Atrial septal defect, sagittal craniosynostosis
COG2010/ DDD283406	missense	c.1684T>C	p.(Cys562Arg)	de novo	nk	nk	nk	9.5	1.7	0.3/5.1yrs	1.0/5.1yrs	mod	no	yes	no	no	no	Mild tonsillar ectopia
COG2003	missense	c.1743G>C	p.(Trp581Cys)	de novo	-1.0	nk	nk	20.3	1.1	1.1	1.2	sev	no	yes	yes	no	yes	Cryptorchidism, lipoma, hirsutism
COG2013/ DDD265343	missense	c.1743G>T	p.(Trp581Cys)	de novo	0.7	nk	2.3	2.5	2.5	2.7	1.4	mod	no	yes	yes	no	yes	Chiari malformation and ventriculomegaly, umbilical hernia
COG2002	missense	c.1748G>A	p.(Cys583Tyr)	de novo	2.5	nk	1.1	15.4	1.7	1.6	1.2	sev	regression	yes	yes	yes	yes	Seizures (tonic-clonic)
COG0510	stop gain	c.1803G>A	p.(Trp601X)	de novo	2.9	nk	1.5	18.8	2.1	0.6	4.1	sev	obsessive	yes	no	no	no	Endochrondroma
COG1972	splice site	c.1851+3G>C		de novo	1.3	nk	1.7	6.6	4.0	-1.2	3.1	mod	no	yes	no	yes	no	Strabismus, myopia, thyroid cyst
COG0553	missense	c.1943T>C	p.(Leu648Pro)	de novo	-0.4	nk	nk	19.0	2.5	3.1	4.3	mild	ASD	no	no	no	no	
COG2021	frameshift	c.2056delG		de novo	0.8	1.8	0.8	10.0	0.6	2.0	0.7	mild	no	nk	no	no	yes	Seizures
COG1942	missense	c.2094G>C	p.(Trp698Cys)	de novo	0.4	nk	nk	21.0	3.7	2.5	1.4/18.9yrs	mod	ASD, severe psychosis and bipolar disorder	yes	yes	yes	no	Menorrhagia, severe constipation
COG1688	missense	c.2099C>T	p.(Pro700Leu)	de novo	1.2	nk	0.4	15.4	2.6		3.3	mod	ASD	yes	yes	yes	no	
COG0316	missense	c.2141C>G	p.(Ser714Cys)	de novo	1.2	nk	nk	4.4	3.0	1.4	2.9	sev	no	yes	yes	yes	no	Bilateral hydroureteronephrosis and left ureteral ectasia, platelet disorder, thick skull vault and sclerosis of sutures
COG2004	missense	c.2204A>C	p.(Tyr735Ser)	de novo	1.6	nk	nk	20.0	2.5	2.8	2.5	mild	no	no	no	no	no	AML-FAB type M4 diagnosed age 12 years
COG0447	missense	c.2207G>A	p.(Arg736His)	de novo	1.0	nk	0.6	8.5	3.0	2.0	2.5	mild	no	yes	no	no	no	
COG1695	missense	c.2245C>T	p.(Arg749Cys)	de novo	0.8	0.6	2.0	15.5	2.8	3.8	1.4	mod	no	yes	no	yes	no	Vesico-ureteric reflux, hypodontia
COG2005	missense	c.2245C>T	p.(Arg749Cys)	de novo	-1.0	nk	0.4	23.0	0.5		2.7	mod	ASD, psychosis and schizophrenia	yes	no	no	no	
COG0108	missense	c.2246G>A	p.(Arg749His)	de novo	0.3	nk	nk	20.8	1.2	1.3	4.4	mod	no	yes	yes	no	no	
COG1632/ DDD263319	in-frame deletion	c.2255_2257delTCT		de novo	1.8	2.2	2.5		nk	nk	nk	mod	no	nk	no	no	no	Tight achilles tendons
COG1512	frameshift	c.2297dupA		de novo	4.0	3.5	nk	13.3	3.8	1.5	1.9	mod	no	yes	no	no	no	
COG2011	missense	c.2309C>T	p.(Ser770Leu)	de novo	0.9	nk	nk	16.3	2.6	-0.1	0.4	mod	Bipolar disorder	yes	yes	yes	no	Aortic root enlargement and mitral valve regurgitation, hyperthyroidism
COG1971	missense	c.2312G>A	p.(Arg771Gln)	de novo	1.2	nk	nk	3.1	3.4	3.4/2.6yrs	3.1	mod	ASD	nk	yes	no	no	Keratosis pilaris
COG1964	missense	c.2401A>G	p.(Met801Val)	de novo	3.0	2.8	2.6	8.8	2.1	-0.2	2.0	mod	regression	yes	nk	yes	yes	
COG1771	missense	c.2512A>G	p.(Asn838Asp)	de novo	0.8	nk	1.5		nk	nk	nk	mild	no	yes	nk	yes	yes	Testicular atrophy
COG1923	missense	c.2644C>T	p.(Arg882Cys)	de novo	3.0	4.4	nk	5.8	-0.2	2.5	1.1	mod	no	yes	yes	no	no	Hydrocephalus secondary to neonatal intraventricular bleed, swallowing difficulties
COG1945	missense	c.2644C>T	p.(Arg882Cys)	de novo	0.8	0.5	0.6	2.0	2.7	0.3	2.9	mod	no	no	yes	no	no	Cryptorchidism, capillary malformation, strabismus, bilateral inguinal herniae, ventriculomegaly
COG1999	missense	c.2644C>T	p.(Arg882Cys)	de novo	0.9	nk		2.0	0.9	2.1	2.2	mod	no	yes	yes	no	no	Ventriculomegaly, obstructive and central sleep apnoea, cryptorchidism
COG2012	missense	c.2645G>A	p.(Arg882His)	de novo	0.3	2.2	1.2	1.5	-0.2	-0.8	-1.4	mod	no	yes	yes	yes	no	Atrial septal defect, bifid sternum, umbilical hernia
COG1760	stop gain	c.2675C>A	p.(Ser892X)	de novo	0.9	1.2	0.4	12.9	4.2	3.0	3.4	mild	no	no	no	no	no	Pes planus
COG0109	missense	c.2705T>C	p.(Phe902Ser)	de novo	1.7	nk	2.0	21.5	1.5	1.4	1.7	mod	ASD	yes	no	yes	no	Mitral and tricuspid regurgitation, polycystic ovarian syndrome, myopia
COG1677	missense	c.2711C>T	p.(Pro904Leu)	de novo	0.7	nk		7.3	3.9	-0.4	3.9	mod	ASD	yes	yes	no	no	Gowers manoeuvre on standing
COG1887	missense	c.2711C>T	p.(Pro904Leu)	de novo	1.8	nk	0.0	9.5	-0.3	0.3	-1.1	mod	Anxiety and ADHD	yes	yes	yes	no	Mitral regurgitation, Chiari malformation
COG1813	gene del			de novo	1.0	1.6	1.5	23.0	3.0	3.2	4.0	mod	no	yes	no	no	no	Double teeth, recurrent infections, polycystic ovaries syndrome
COG1961	gene del			de novo	-0.1	nk	nk	5.8	2.7	1.9	2.8	mod	ASD	no	yes	no	no	Patent ductus arteriosus, hirsutism
COG2006	gene del			de novo	-1.1	nk	nk	5.8	2.3	1.6	2.1	mod	ASD	no	yes	no	no	Patent ductus arteriosus, hirsutism
COG2014	gene del			de novo	0.3	0.8	0.2	3.0	2.2	0.7/2.0yrs	2.8	mild	ASD, regression	no	no	no	yes	Recurrent ear infections, subclinical seizures

Abbreviations: nk, not known; ID, intellectual disability; CAL, café au lait; SD, standard deviation; gene del, whole gene deletion; BW, birth weight; BHC, birth head circumference; BL, birth length; Ht, height; Wt, weight; HC, head circumference; mod, moderate; sev, severe; ASD, autistic spectrum disorder; br MRI, brain magnetic resonance imaging; AML, acute myeloid leukaemia; FAB, Franco-American-British; ADHD, attention deficit hyperactivity disorder; AVNRT, atrio-ventricular nodal re-entry tachycardia

## Results

**Figure 2.**
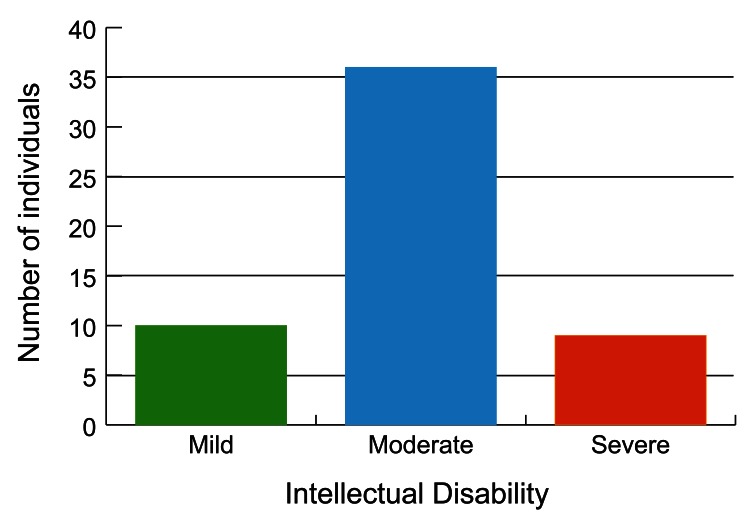
Graph showing the range of intellectual disability in TBRS.

All 55 individuals had an intellectual disability: 18% had a mild intellectual disability (10/55); 65% had a moderate intellectual disability (36/55) and 16% had a severe intellectual disability (9/55) ( Table 1, Figure 2). Behavioural/psychiatric issues were reported in 51% (28/55) individuals and included combinations of autistic spectrum disorder (20 individuals); anxiety (three individuals); neurodevelopmental regression (four individuals two of whom regressed in teenage years); psychosis/schizophrenia (three individuals); aggressive outbursts (two individuals), and bipolar disorder (two individuals) ( Table 1).

**Figure 3.**
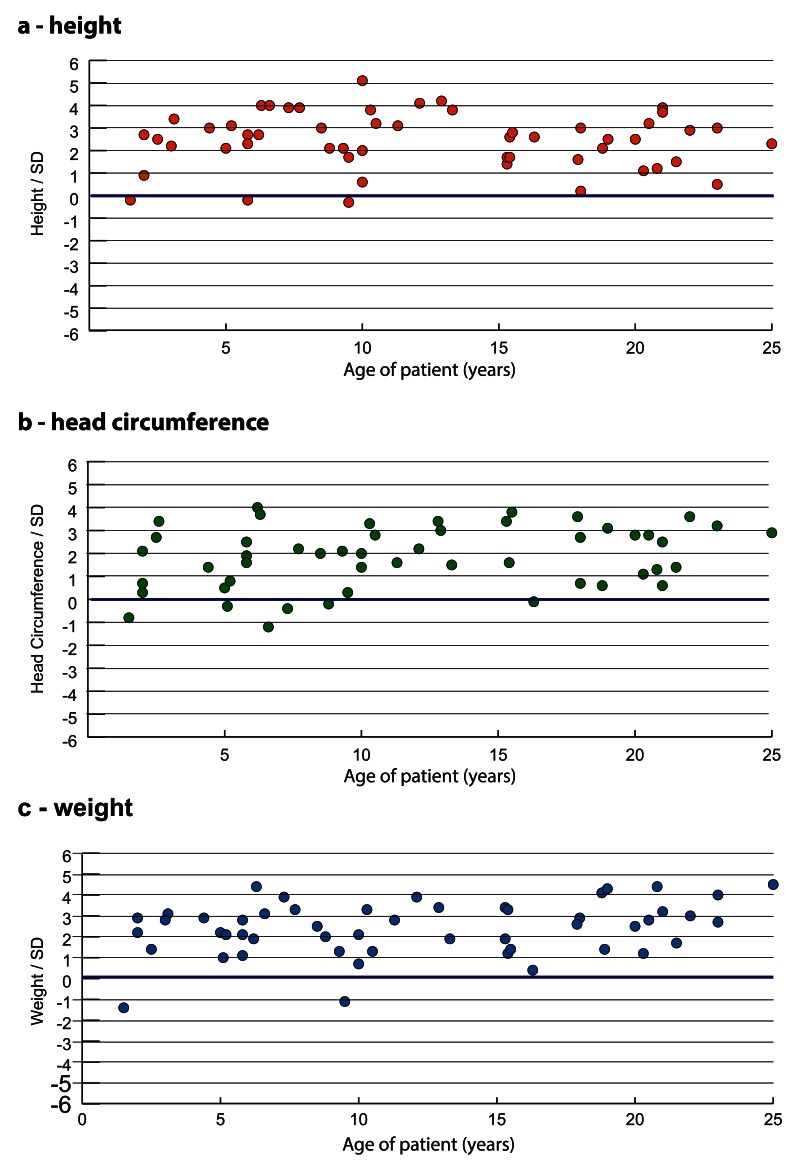
Growth profile in individuals with TBRS **a**) height, **b**) head circumference and **c**) weight. The blue line represents the mean.

Postnatal overgrowth (defined as height and/or head circumference at least two standard deviations above the mean (≥2SD) ^2, 13^, was reported in 83% (44/53) individuals. Obesity, with a weight ≥2SD, was reported in 67% (34/51). The range of individual postnatal heights, head circumferences and weights is shown in Table 1 and Figure 3. The mean birth weight was 1.3SD with a range from -1.1 to 4.0 SD. We had limited data for birth head circumference and birth length, but their mean was 2.3SD and 1.6SD, respectively.

**Figure 4.**
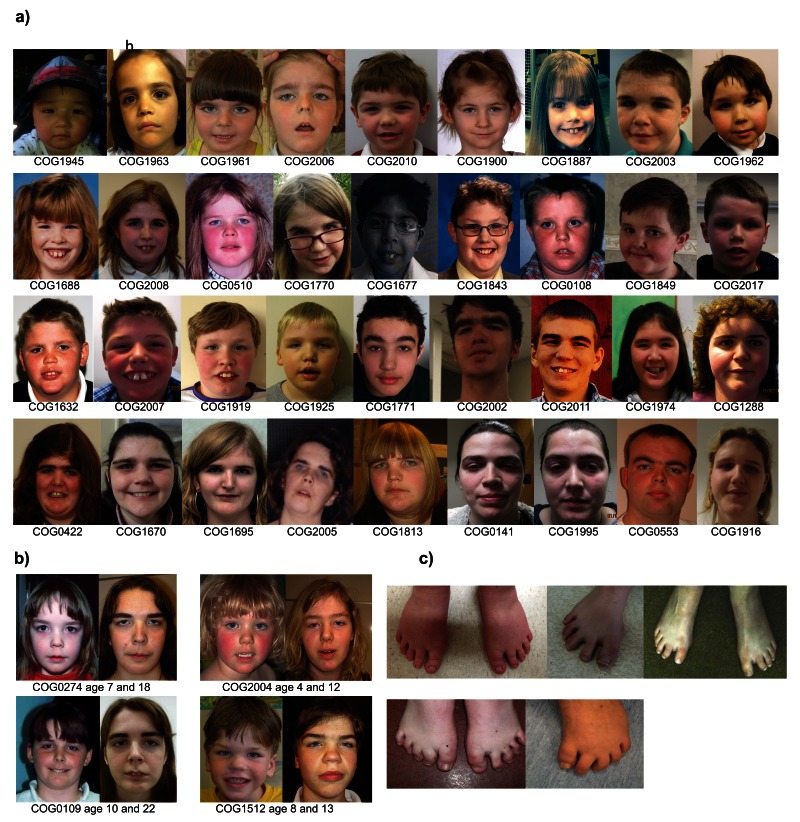
**a**) The facial appearance of children and adults with TBRS; **b**) the evolving facial appearance in four individuals with TBRS; and **c**) the characteristic short, widely spaced toes seen in TBRS.

There were some shared, but subtle, facial characteristics often only becoming apparent in early adolescence ( Figure 4a and b). These included low-set, horizontal thick eyebrows; narrow palpebral fissures; coarse features and a round face. The two upper central incisors were also frequently enlarged and prominent.

Additional clinical features reported in greater than 20% (≥ 11) individuals included: joint hypermobility (74%, 37/50); hypotonia (54%, 28/52); kyphoscoliosis (33%, 18/55) and afebrile seizures (22%, 12/55) ( Table 1). In addition, short, widely spaced toes were frequently mentioned, but the overall frequency is unclear as we did not specifically ask about feet/toes on the clinical proforma ( Figure 4c).

Clinical features reported in at least two but fewer than 20% individuals included cryptorchidism (six individuals); ventriculomegaly (four individuals) and Chiari malformation (three individuals). In addition, a range of cardiac anomalies (including atrial septal defect, mitral/tricuspid valve incompetence, patent ductus arteriosus, aortic root enlargement and atrio-ventricular re-entry tachycardia) were reported in nine individuals. However, of note, two individuals with cardiac anomalies (patent ductus arteriosus, COG1961 and COG2006) were identical twins with *DNMT3A* whole gene deletions encompassing >40 genes. The patent ductus arteriosus in these individuals may, therefore, be attributable to twinning, alternative genes in the deleted region or the combined effect of a number of deleted genes.

Acute myeloid leukaemia (AML), AML-FAB (French-American-British classification) type M4, was diagnosed in one individual at the age of 12 years (COG2004). This individual had a *de novo* heterozygous c.2204A>C p.(Tyr735Ser) *DNMT3A* variant, identified in DNA obtained seven years prior to the diagnosis of AML.

Full clinical details from the 55 individuals are provided in Table 1.

## Discussion

We have evaluated clinical data from 55 individuals with *de novo* constitutive *DNMT3A* variants to define the phenotype of TBRS. An intellectual disability (most frequently in the moderate range) and overgrowth (defined as height and/or head circumference ≥2SD above the mean) were reported in ≥80% of individuals and have been designated major clinical associations. Frequent clinical associations, reported in 20–80% of individuals with constitutive *DNMT3A* variants, included joint hypermobility, obesity, hypotonia, behavioural/psychiatric issues (most frequently autistic spectrum disorder), kyphoscoliosis and afebrile seizures. In addition, many individuals had a characteristic facial appearance although this may only be recognizable in adolescence.

TBRS overlaps clinically with other OGID syndromes including Sotos syndrome ( OMIM 117550), Weaver syndrome ( OMIM 277590), Malan syndrome ( OMIM 614753) and the OGID syndrome due to *CHD8* gene variants ^2^. However, TBRS is more frequently associated with increased weight than the other OGID syndromes and may be distinguishable through recognition of the associated facial features, and absence of the facial gestalt of other OGID syndromes.

Somatic *DNMT3A* variants are known to drive the development of adult AML and myelodysplastic syndrome and over half of the *DNMT3A* somatic variants target a single residue, the p.Arg882 residue ^14– 17^. AML, diagnosed in childhood, has now been identified in two individuals with (likely) constitutive *DNMT3A* variants from a total of 77 (1/55 individuals in the current study and 1/22 previously reported individuals) ^7^. One of these individuals had a *de novo* c.2644CT p.(Arg882Cys) *DNMT3A* variant and developed AML at 15 years of age ^7^. The variant was present in genomic DNA extracted from the patient’s remission blood sample and skin fibroblasts. The second individual had a c.2204A>C p.(Tyr735Ser) *DNMT3A* variant identified in DNA obtained at 5 years of age and developed AML at the age of 12 years. Whilst these data indicate that AML may be a rare association of TBRS, currently the numbers of individuals reported with TBRS and AML are too few to either accurately quantify the risk of AML in TBRS or determine whether this risk is influenced by the underlying *DNMT3A* genotype. Further studies are required to address this.

The majority of individuals with TBRS are healthy and do not require intensive clinical follow up. However, our practice is to inform families and paediatricians of the possible TBRS complications of behavioural/psychiatric issues, kyphoscoliosis and afebrile seizures to introduce a low threshold for their investigation and/or management. In addition, we undertake a baseline echocardiogram at initial diagnosis to investigate cardiac anomalies detectable on ultrasound scan and frequently refer patients to physiotherapy to evaluate the degree of hypotonia and/or joint hypermobility and to determine whether targeted exercises may be beneficial. Finally, in the absence of evidence-based surveillance protocols for haematological malignancies, we advise clinical vigilance for symptoms possibly related to a haematological malignancy such as easy bruising, recurrent bleeding from gums or nosebleeds, persistent tiredness and recurrent infections.

## Ethics and consent

The study was approved by the London Multicentre Research Ethics Committee (MREC MREC/01/2/44).

Written informed consent was obtained from participants and/or parents for participation in the study (n=55) and publication of photographs of participants shown in Figure 4 (n=41).

## Data availability

All data underlying the results are available as part of the article and no additional source data are required.

## Supplementary material

Supplementary Table 1: Computational evaluation of *DNMT3A* missense variants.

.

Supplementary File 1: A full list of all the collaborators, study participants and the clinicians that recruited them, in this study.
