# Supplementary material for: The Tatton-Brown-Rahman Syndrome: A clinical study of 55 individuals with de novo constitutive DNMT3A variants
Source: Wellcome Open Res. 2018 Apr 23;3:46. [Version 1] doi: 10.12688/wellcomeopenres.14430.1 (PMC5964628; doi:10.12688/wellcomeopenres.14430.1)
Supplement: Supplementary file 1 [file wellcomeopenres-3-15708-s0001.tgz › 89b28d87-cfe9-4a44-9e1d-8d2e1c0c0fa3.docx]

**Supplementary Table 1**

| **Nucleotide change** | **Protein change** | **GnomAD allele count** | **Mutation Taster** | **SIFT** |
| --- | --- | --- | --- | --- |
| c.541C>T | p.(Arg181Cys) | 0 | Disease causing | Damaging |
| c.892G>A | p.(Gly298Arg) | 0 | Disease causing | Damaging |
| c.892G>T | p.(Gly298Trp) | 0 | Disease causing | Damaging |
| c.901C>T | p.(Arg301Trp) | 0 | Disease causing | Damaging |
| c.929T>A | p.(Ile310Asn) | 0 | Disease causing | Damaging |
| c.1154C>T | p.(Pro385Leu) | 7 | Disease causing | Damaging |
| c.1523T>C | p.(Leu508Pro) | 0 | Disease causing | Damaging |
| c.1594G>A | p.(Gly532Ser) | 0 | Disease causing | Damaging |
| c.1643T>A | p.(Met548Lys) | 0 | Disease causing | Damaging |
| c.1643T>C | p.(Met548Thr) | 0 | Disease causing | Damaging |
| c.1645T>C | p.(Cys549Arg) | 0 | Disease causing | Damaging |
| c.1684T>C | p.(Cys562Arg) | 0 | Disease causing | Damaging |
| c.1743G>C | p.(Trp581Cys) | 2 | Disease causing | Damaging |
| c.1748G>A | p.(Cys583Tyr) | 2 | Disease causing | Damaging |
| c.1943T>C | p.(Leu648Pro) | 0 | Disease causing | Damaging |
| c.2094G>C | p.(Trp698Cys) | 0 | Disease causing | Damaging |
| c.2099C>T | p.(Pro700Leu) | 1 | Disease causing | Damaging |
| c.2141C>G | p.(Ser714Cys) | 2 | Disease causing | Damaging |
| c.2204A>C | p.(Tyr735Ser) | 3 | Disease causing | Damaging |
| c.2207G>A | p.(Arg736His) | 11 | Disease causing | Damaging |
| c.2245C>T | p.(Arg749Cys) | 7 | Disease causing | Damaging |
| c.2246G>A | p.(Arg749His) | 5 | Disease causing | Damaging |
| c.2309C>T | p.(Ser770Leu) | 6 | Disease causing | Damaging |
| c.2312G>A | p.(Arg771Gln) | 1 | Disease causing | Damaging |
| c.2401A>G | p.(Met801Val) | 4 | Disease causing | Damaging |
| c.2512A>G | p.(Asn838Asp) | 3 | Disease causing | Damaging |
| c.2644C>T | p.(Arg882Cys) | 35 | Disease causing | Damaging |
| c.2645G>A | p.(Arg882His) | 62 | Disease causing | Damaging |
| c.2705T>C | p.(Phe902Ser) | 1 | Disease causing | Damaging |
| c.2711C>T | p.(Pro904Leu) | 9 | Disease causing | Damaging |
