# Supplementary material for: The Tatton-Brown-Rahman Syndrome: A clinical study of 55 individuals with de novo constitutive DNMT3A variants
Source: Wellcome Open Res. 2018 Apr 23;3:46. [Version 1] doi: 10.12688/wellcomeopenres.14430.1 (PMC5964628; doi:10.12688/wellcomeopenres.14430.1)
Supplement: Supplementary file 2 [file wellcomeopenres-3-15708-s0000.tgz › 0c24a51e-d7d2-4c54-b15f-3c5ecd3ed6be.docx]

**Supplementary File 1**

The Childhood Overgrowth Collaboration

The following individuals coordinated recruitment and collection of the families and samples. M-C. Addor, M. Akgul, L. Aksglaede, M. Ahmed, D. Amor, K. Anderson, R. Anderson, S. Andries, H. Archer, R. Armstrong, P. Ashton-Prolla, M. Bahceci, M. Balasubramanian, D. Baralle, D. Barge, A. Barnicoat, M. Barrow, J. Barwell, G. Baujat, G. Baynam, P. Beales, K. Becker, E. Beckh-Arnold, A. Ben-Yehuda, J. Berg, B. Bernhard, S. Bhal, M. Bhat, J. Birch, L. Bird, M. Bitner-Glindzicz, E. Blair, J. Bliek, M. Blyth, A. Bottani, M. Bouma, M. Boxill, F. L. Bradley, A. Brady, Breatnach, G. Brice, B. Buehler, A. Burke, J. Burn, J. Campbell, N. Canham, B. Castle, K. Chandler, R. Chandrasena, E. Chang, C. Christenden, C. Chu, D. Cilliers, A. Clarke, J. Clayton-Smith, C. Clericuzio, V. Clowes, T. Cole, A. Colley, A. Collins, F. Connell, J. Cook, I. Cordeiro, E. Crocker, Y. Crow, V. Culic, T. Cushing, T. Dabir, A. Dalton, S. Danda, R. Davidson, S. Davies, R. Day, D. Dearnaley, M-A. Delrue, M. De Roy, V. de Soberanis, M. de Ville, N. Dennis, C. Deshpande, B. Desouza, L. Devlin, A A. Dieckmann, -M. Differ, R. Dinwiddie, A. Dixit, A. Dobbie, J. Dominguez, A. Donaldson, D. Donnai, D. Donnelly, H. Dorkins, M. Doz, J. Dupont, D. Eastwood, M. Edwards, I. Ellis, F. Elmslie, L. Escobar, R. Evans, F. Faravelli, C. Fauth, H. Firth, R. Fisher, T. Fiskerstrand, D. Fitzpatrick, A. Flanagan, F. Flinter, P. Foley, A. Foster, N. Foulds, W. Foulkes, J. Franklin, A. Fryer, H. Fryssira, A. Gallagher, S. Garcia, C. Gardiner, M. Gardner, C. Garrett, B. Gener, M. Gerrard, R. Gibbons, Y. Gillerot, H. Goel, D. Goudie, K. Gowrishankar, C. Graham, A. Green, N. Gregersen, J. Hale, M. Hamilton, J. Harper, R. Harrison, V. Harrison, A. Henderson, P. Henman, R. Hennekam, E. Hobson, S. Hodgson, M. Holder, S. Holder, T. Homfray, D. Horovitz, H. Hughes, Z. Huma, M. Hunter, J. Hurst, W-L. Hwu, A. Irvine, M. Irving, L. Izatt, M-L. Jacquemont, S. Jagadeesh, L. Jenkins, U. Jensen, C. Jessen, D. Johnson, J. Johnson, E. Jones, L. Jones, A. Jorgensen, D. Josifova, S. Joss, Dr. Kanabar, P. Kannu, K. Keppler-Noreuil, B. Kerr, H. Kingston, J. Kingston, U. Kini, E. Kinning, A. Krause, V. Krishnamurthy, A. Kumar, D. Kumar, A. Medeira, V. Meiner, C. Mercer, K. Milstein, Y. Miyoshi, E. Moran, K. Lachlan, W. Lam, P. Lapunzina, M. Lees, N. Leonard, G. Levitt, I. Lewis, J. Liebelt, A. Livesey, C. Longman, T. Lopponen, Dr Lozano, A. Lucassen, P. Lunt, S-A Lynch, S. Lyonnet, J. MacDonnell, A. Magee, E. Maher, S. Maitz, A. Male, S. Mansour, C. Marcelis, E. McCann, V. McConnell, T. McDevitt, M. McEntagart, J. McGaughran, G. McGillivray, R. McGowan, S. McKee, C. McKeown, C. Meany, S. Mehta, K. Metcalfe, Z. Miedzybrodzka, S. Mohammed, G. Monaghan, T. Montgomery, A. Morgan, B. Morland, P. Morrison , J. Morton, R. Mudgal, A. Munaza, V. Murday, S. Nampoothiri, K. Nathanson, K. Neas, A. Nemeth, G. Neri, R. Newbury-Ecob, C. Nur Semerci, C. Ockeloen, C. Oley, C. Owen, K. Ozono, Panarello, S-M. Park, M. Parker, C. Patel, M. Patton, S. Payne, M. Pearson, J. Piard, D. Pilz, M. Pinkney, B. Plecko, M. Pocha, G. Poke, R. Posmyk, C. Pottinger, K. Prescott, S. Price, K. PritchardJones, A. Proctor, V. Puthi, O. Quarrell, A. Raas-Rothchild, E. Rahikkala, W. Raith, J. Rankin, L. Raymond, G. Rea, L. Read, W. Reardon, E. Reid, H.Rees, N. Revencu, O. Rittinger, M. Robards, A. Roposch, E. Rosser, D. Rourke, D. Ruddy, A. Saggar, N. Saleh, V. Saletti, J. Sampson, R. Sandford, H. Santos, A. Sarkar, R. Scott, I. Scurr, C. Searle, A. Selicorni, R. Semple, S. Sharif, A. Shaw, C. Shaw-Smith, D. Shears, J. Shelagh, N. Shur, L. Side, M. Simon, F. Skovby, G. Smith, S. Smithson, M. Splitt, M. Stevens, A. Stewart, F. Stewart, H. Stewart, K. Stopps, C. Stumpel, K. Stuurman, D. Subramanian, M. Suri, A. Swain, E. Sweeney, K. Szakszon, Y. Sznajer, G. Tanateles, A. Taylor, C. Taylor, M. Teixeira, I.K. Temple, E. Thomas, E. Thompson, F. Thonney, M. Tischowitz, J. Tolmie, S. Tomkins, S. Turkmen, A. Turner, P. Turnpenny, M. Van-Haelst, L. Van Maldergem, P. Vasudevan, I. Veenstra-Knol, C. Verellen, I.C. Verma, J. Vigneron, E. Wakeling, L. Wainwright L. Walker, D. Weaver, P. Wheeler, K. White, S. White, M. Whiteford, D. Williams, L. Wilson, R. Winter, G. Woods, M. Wright, N. Yachelevich, A. Yeung, A. Zankl
