# Supplementary material for: The Tatton-Brown-Rahman Syndrome: A clinical study of 55 individuals with de novo constitutive DNMT3A variants
Source: Wellcome Open Res. 2018 Apr 23;3:46. [Version 1] doi: 10.12688/wellcomeopenres.14430.1 (PMC5964628; doi:10.12688/wellcomeopenres.14430.1)
Supplement: Peer review history

# Peer review: Reviewer response for version 1

**DOI:** 10.21956/wellcomeopenres.15708.r33053

**Published:** 2018-05-29

**Authors:** Wei Shen

**Affiliations:** 1 ARUP Laboratories, Salt Lake City, UT, USA; 2 University of Utah, Salt Lake City, UT, USA

In this very well written manuscript, the authors described the largest cohort of patients with the Tatton-Brown-Rahman syndrome (TBRS) to date, and further delineated the clinical phenotype associated with TBRS. It would be very interesting to explore any genotype-phenotype correlations in this cohort combined with other patients reported in the literature if needed. For example, the individuals without overgrowth in this cohort all had missense variants, whereas all patients with clearly loss-of-function variants including truncating (nonsense and frame-shift) variants or gene-deletions exhibited overgrowth. While the functional consequences of Arg882 missense variants (p.Arg882His and p.Arg882Cys) were investigated in both somatic and germline settings (Spencer DH et al. Cell 2017, Russler-Germain et al. Cancer Cell 2014), the effects of other missense variants on DNMT3A function are still unclear (presumably loss-of-function). It would be also interesting to see how many of the DNMT3A germline variants reported here were also observed as somatic mutations in leukemia.

 Minor points:

1. Please describe the protein changes for the indel variants in Table 1 according to syntax recommended by HGVS.
2. Please change “c.2644CT” to “c.2644C>T” in the top line on page 11.
3. Was any patient (other than the 13 patients first reported in the 2014 Nat Genet paper) previously reported? If so, please reference the original publication.  

Is the work clearly and accurately presented and does it cite the current literature?

Yes

If applicable, is the statistical analysis and its interpretation appropriate?

Not applicable

Are all the source data underlying the results available to ensure full reproducibility?

Yes

Is the study design appropriate and is the work technically sound?

Yes

Are the conclusions drawn adequately supported by the results?

Yes

Are sufficient details of methods and analysis provided to allow replication by others?

Yes

Reviewer Expertise:

NA

I confirm that I have read this submission and believe that I have an appropriate level of expertise to confirm that it is of an acceptable scientific standard.

# Peer review: Reviewer response for version 1

**DOI:** 10.21956/wellcomeopenres.15708.r33035

**Published:** 2018-05-18

**Authors:** William T. Gibson, Sharri Cyrus

**Affiliations:** 1 Diabetes Research Group, BC Children's Hospital Research Institute, Vancouver, BC, Canada; 2 Department of Medical Genetics, University of British Columbia, Vancouver, BC, Canada

This is a very well written article, which expands on the previously-reported phenotype and recommends management guidelines for a rare and recently-described syndrome. The inclusion of multiple patient photos and clinical details will be quite helpful for other physicians who have one or more patients with rare variants in this gene. Similarly, the aggregation of the rare variants with clinical annotations will assist clinical diagnostic labs in the interpretation of rare variants they encounter in NGS panels, clinical exomes and whole genomes.

 The authors mention “joint hypermobility” as a feature, but do not offer additional details. In clinical practice, one frequently encounters patients who claim to have joint hypermobility (or to have had it in the past), yet the degree of hypermobility and the number of joints affected varies greatly from patient to patient. Thus, the phenotypic spectrum of “joint hypermobility” can vary, from minor painless hyperextensibility of the small joints of the hands in childhood all the way to significantly increased range of motion among both large and small joints that persists into adulthood. A full assessment of the Beighton scale and of range-of-motion of the other joints is not likely to have been documented by all referring clinicians, but perhaps the column on “Joint hypermobility” could be split into two columns such as “Joint hypermobility – history” (for patients who report it as a symptom) and “Joint hypermobility – demonstrated” (for patients in whom hypermobility is documented as a sign on physical exam). Alternatively, the categories “nk” “no” and “yes” could be adjusted to “nk” “no” “yes (hist)” and “yes (exam)” or something similar.

 Many of the facial photos presented appear to show downslanted palpebral fissures, yet the authors comment only on “narrow palpebral fissures” in the article. Do the authors have enough data to comment on this feature, and/or could they have the available facial photographs evaluated systematically for this feature? It is likely to be some time before another cohort of this size or larger is assembled and published, so it may be worthwhile to investigate this aspect of the facial gestalt in a little more detail. It would also be helpful for the authors to comment on the presence or absence of hypertelorism, as some dysmorphologists consider an interpupullary distance greater than the 97 ^th^ %ile for age to be a useful sign in the assessment of OGID, whereas others “adjust” the eye spacing in light of the head circumference (which is frequently >+2SD for age in OGID).

 Minor Spelling and Grammatical Errors:

 In the abstract, the authors state “weight ^3^2SD” – perhaps they mean “weight >+2SD” or “weight Z-score +2 or higher”?

 In the abstract, “TBRS” should be followed by a period.

Is the work clearly and accurately presented and does it cite the current literature?

Yes

If applicable, is the statistical analysis and its interpretation appropriate?

Not applicable

Are all the source data underlying the results available to ensure full reproducibility?

Yes

Is the study design appropriate and is the work technically sound?

Yes

Are the conclusions drawn adequately supported by the results?

Yes

Are sufficient details of methods and analysis provided to allow replication by others?

Yes

Reviewer Expertise:

OGID, PRC2 Complex, Epigenetic risk factors for rare diseases, Intracranial Aneurysms

We confirm that we have read this submission and believe that we have an appropriate level of expertise to confirm that it is of an acceptable scientific standard.

# Peer review: Reviewer response for version 1

**DOI:** 10.21956/wellcomeopenres.15708.r32979

**Published:** 2018-05-02

**Authors:** Emma L. Wakeling

**Affiliations:** 1 North West Thames Regional Genetics Service, London North West University Healthcare NHS, London, UK

This is a concise and well-written paper summarising the clinical phenotype in 55 patients with Tatton-Brown-Rahman syndrome due to de novo constitutive DNMT3A variants.The findings are clearly presented with the use of figures and detailed clinical information in table 1.

 Minor comments are as follows:

1. The abstract list of frequent associations should be slightly re-punctuated for clarity: ‘behavioural/psychiatric issues, most frequently autistic spectrum disorder (51%);’
2. There is no indication of the male: female ratio within the cohort. This is relevant to the frequency of cryptorchidism in affected males.
3. Although increased weight (≥2 SD) is clearly a feature (Figure 3), this is in the context of overgrowth (height and/or head circumference ≥2 SD). It would be helpful to know the frequency of true obesity (BMI ≥ 30) and to make this distinction in the paper.
4. Although the focus of the paper is a clinical description of TBRS, it would be helpful to discuss briefly the clustering of missense and in-frame deletions (with two exceptions) within the three DNMT3A domains and possible genotype-phenotype correlation (this is only mentioned in the context of AML).

Is the work clearly and accurately presented and does it cite the current literature?

Yes

If applicable, is the statistical analysis and its interpretation appropriate?

Not applicable

Are all the source data underlying the results available to ensure full reproducibility?

Yes

Is the study design appropriate and is the work technically sound?

Yes

Are the conclusions drawn adequately supported by the results?

Yes

Are sufficient details of methods and analysis provided to allow replication by others?

Yes

Reviewer Expertise:

NA

I confirm that I have read this submission and believe that I have an appropriate level of expertise to confirm that it is of an acceptable scientific standard.
